# Static Estimation of Vista-Space Egocentric Distance with Iterative Feedback: A Cognitive–Perceptual Task

**DOI:** 10.3390/life16010173

**Published:** 2026-01-21

**Authors:** Constantin Ciucurel, Elena Ioana Iconaru

**Affiliations:** Department of Medical Assistance and Physical Therapy, University Center of Pitesti, National University of Science and Technology Politehnica Bucharest, 110040 Pitesti, Romania; constantin.ciucurel@upb.ro

**Keywords:** perceptual adaptation, spatial accuracy, feedback-based learning, cognitive calibration, distance judgment, mobile web application

## Abstract

Accurate egocentric distance estimation in vista space depends on the interaction between perceptual encoding and cognitive recalibration. This study examined how iterative, feedback-based learning modulates spatial accuracy, perceptual bias, and task efficiency in large-scale environments. A total of 133 participants (mean age = 26.3 ± 7.44 years) performed distance estimations on three outdoor targets (134 m, 575 m, 1463 m) using a mobile web application providing immediate corrective feedback (too short/too long). Six variables were analyzed: first estimate (FE), error of first estimate (EFE), mean estimate (ME), error of mean estimate (EME), number of attempts (NAs), and trial duration (TD). Given the non-normal data distribution, nonparametric tests were applied (Friedman and Wilcoxon signed-rank tests with Bonferroni correction). All variables showed significant within-subject effects across distances (*p* < 0.001). Post hoc analyses indicated that EFE and EME differed significantly between all target pairs (*p* < 0.0167), revealing a shift from slight overestimation at 134 m to increasing underestimation at 575 m and 1463 m. NA was significantly higher for the farthest target (*p* < 0.0167), indicating greater cognitive load and iterative correction effort. TD differed significantly only between consecutive distances (*p* < 0.0167), suggesting non-linear temporal adaptation. These results demonstrate that iterative feedback improves perceptual stability and efficiency but does not remove distance compression. The consistent bias and adaptive response patterns support a feedback-driven, binary search-like recalibration mechanism. The proposed mobile paradigm offers a scalable and valid approach for assessing perceptual–cognitive calibration in both natural and virtual spatial contexts.

## 1. Introduction

Perceiving spatial distances accurately is a fundamental component of human space mobility and environmental interaction. Egocentric distance estimation, the ability to judge how far objects are from oneself, relies on the integration of multiple sensory cues and cognitive processes. Egocentric distance perception in static conditions, where both the observer and target remain stationary, primarily relies on visual cues such as angular declination, relative size, and texture gradients [1]. Unlike dynamic contexts involving motion parallax and spatial updating, the static state isolates perceptual and cognitive mechanisms of distance estimation [2,3].

Generally, perceptual judgments of distance are prone to systematic biases, especially in large-scale or vista space environments where direct action-based feedback is limited. In the context of large-scale environments, distance estimation occurs within vista space, a perceptual domain extending beyond approximately 30 m. Within this range, spatial judgments rely primarily on visual perspective and cognitive calibration rather than on direct physical interaction with the environment [4]. Similar perceptual biases have also been observed in virtual environments, where egocentric distances are frequently underestimated, affecting spatial judgments even when no real-world reference exists [5].

In experimental paradigms addressing egocentric distance perception under naturalistic conditions, action-based measures provide a more direct assessment of spatial accuracy compared to purely perceptual judgments. Studies of distance perception in the real environment typically use visually directed action tasks in which participants carry out actions toward previously seen targets [6]. The gold standard in this area of research is blindfolded walking, in which participants view a target and then immediately attempt to walk to the target while blindfolded [7]. This paradigm minimizes visual feedback and emphasizes the internal representation of spatial metrics, thereby isolating the contribution of proprioceptive and vestibular cues to distance updating. Such tasks allow researchers to quantify the fidelity of internal spatial representations and to distinguish between perceptual encoding errors and motor execution biases in distance estimation [8].

However, locomotor-based estimates show marked limitations in large-scale or vista-space contexts. At distances exceeding approximately 100 m, verbal estimations tend to remain relatively accurate, whereas locomotor estimates have been found to be grossly underestimated [9]. This discrepancy underscores the reduced reliability of motor-based spatial updating when physical interaction with distant targets is no longer ecologically meaningful, reinforcing the importance of perceptual and cognitive calibration mechanisms in long-range distance estimation.

Recent research in perceptual learning has emphasized that feedback and iterative recalibration can substantially improve spatial accuracy [10,11]. When individuals receive trial-by-trial information about the correctness of their estimates, perceptual and cognitive systems adjust their internal representations, leading to a process known as perceptual adaptation or cognitive calibration. While traditional paradigms, such as blindfolded walking, assess embodied spatial updating through motor execution, feedback-based perceptual tasks offer a complementary approach, focusing on the recalibration of internal distance metrics through cognitive mechanisms rather than locomotor cues. Extending this perspective, informative feedback has been shown to promote perceptual adaptation and modulate behavioral performance, as well as associated neural activation in visual tasks, highlighting its role as a key signal in perceptual plasticity [12]. Additionally, feedback can influence decision strategies and confidence calibration in perceptual judgments, reducing response bias even when sensitivity remains unchanged [13].

With the expansion of web technologies, mobile-based experimental paradigms have become increasingly viable for behavioral research. Such platforms allow for real-time data collection, flexible stimulus presentation, and scalable participant recruitment outside traditional laboratory settings. Recent research emphasizes that spatial cognition is inherently embodied, and systems that actively engage the body, such as tangible and embodied interfaces (TEIs), can enhance spatial skills while extending interaction into physical space and supporting perceptual and cognitive calibration [14]. Applying these principles to mobile-based paradigms enables precise measurement of egocentric distance estimation and promotes cognitive calibration through embodied interactions.

In this context, iterative feedback paradigms provide a valuable framework for exploring the interplay between sensory perception and cognitive regulation. When participants repeatedly estimate a target distance and receive directional feedback (too short/too long), the task becomes not merely perceptual but also cognitive, requiring hypothesis testing, error monitoring, and adaptive recalibration of internal distance metrics. This dual-level engagement positions the procedure as a perceptual–cognitive calibration task, bridging low-level sensory processing with higher-order inferential reasoning [15,16]. Moreover, evidence from the Action-Specific Perception (ASP) framework indicates that perceptual judgments are modulated by task performance variables, underscoring the tight coupling between perception and action, and the critical role of feedback in refining spatial accuracy [17]. Such feedback-driven adaptation has been shown to enhance distance accuracy and reduce perceptual bias across trials, supporting models of active perceptual learning and Bayesian updating in spatial cognition [18,19].

The present study introduces a mobile web application designed to assess static egocentric distance estimation in a controlled yet ecologically valid context. Participants performed multiple iterative estimations of distances to reference objects, receiving immediate feedback to refine their perceptual accuracy across trials. The main objective of this research was to analyze how feedback-driven iteration influences both the precision and efficiency of distance estimation, and to explore individual differences in perceptual–cognitive adjustment mechanisms.

By combining the methodological rigor of controlled perceptual testing with the flexibility of a mobile-based digital platform [20], this study offers an innovative approach to investigating large-scale egocentric distance estimation. The integration of iterative feedback supports the development of adaptive cognitive assessment methods and enhances the applicability of perceptual calibration research to both real-world and virtual environments.

## 2. Materials and Methods

### 2.1. Study Design

This cross-sectional exploratory study was designed to investigate static egocentric distance estimation within vista-space conditions using an iterative feedback paradigm implemented through a mobile web application. The experimental framework aimed to analyze how real-time corrective feedback modulates perceptual accuracy and cognitive recalibration across successive estimation trials. By minimizing motor involvement and emphasizing visual–cognitive adjustment processes, the design isolated perceptual encoding mechanisms and adaptive error correction dynamics. Data were collected in controlled outdoor conditions to ensure ecological validity while maintaining standardization of stimulus presentation and response recording.

### 2.2. Participants

Participants were 133 undergraduate students of Caucasian ethnicity, enrolled in Physical Therapy and Occupational Therapy programs at the University Center of Pitești, National University of Science and Technology Politehnica Bucharest. Recruitment was conducted on a voluntary basis, and informed consent was obtained electronically at the time of accessing the experimental web application. Inclusion criteria required normal or corrected-to-normal visual acuity and the absence of neurological, vestibular, or musculoskeletal conditions that could affect spatial perception. Exclusion criteria included uncorrected visual impairments, ocular pathology, cognitive deficits [21], or prior participation in similar distance estimation studies that could influence perceptual adaptation.

### 2.3. Study Size

Based on power calculations conducted a priori using the parametric equivalent of the planned non-parametric repeated-measures design, at least 66 participants were needed to detect a medium effect size (f = 0.20) with a significance level of α = 0.05 (probability of Type I error) and 95% statistical power [22]. The final sample of 133 participants exceeds this requirement, ensuring robust detection of feedback-driven effects, accounting for potential data exclusions, and reflecting the availability of additional participants rather than being determined solely by expected effect sizes.

### 2.4. Experimental Settings

The experimental sessions were conducted from a fixed observation point located at a first-floor window within the university campus. From this position, participants had a clear, unobstructed frontal view of three distinct and easily identifiable large buildings selected as visual targets. The exact distances from the observation point to the targets were measured using a Vortex Diamondback HD 2000 laser rangefinder (Vortex Optics, Barneveld, WI, USA), yielding true distance (D) values of 134 m, 575 m, and 1463 m, respectively, with an accuracy of ±1.83 m for 134 m, and ±2.74 m for 575 m and 1463 m, according to the manufacturer.

All sessions were performed under consistent environmental conditions: clear sky, stable natural lighting, and minimal atmospheric interference, between 10:00 and 12:00 a.m. Participants remained at the same fixed observation point throughout testing, maintaining a constant line of sight toward the designated targets.

### 2.5. Experimental Task and Procedure

The experimental task was implemented as an interactive module within a custom-developed mobile web application, designed to run directly on participants’ smartphones and to record egocentric distance estimations while providing immediate trial-by-trial feedback. Participants accessed the application via a secure web link distributed individually before the testing session, ensuring compatibility with both Android and iOS devices. The platform was developed in JavaScript (ECMAScript 2022 specification) with HTML5 (W3C Recommendation, 2014) and CSS3 (W3C Recommendation, 2018) for the user interface, hosted and version-controlled via GitHub (GitHub Inc., San Francisco, CA, USA), with data stored and synchronized in real time using Google Firebase Realtime Database (Google LLC, Mountain View, CA, USA). This architecture enabled cross-platform deployment and secure, time-stamped data acquisition directly from mobile devices, ensuring integrity and reproducibility of experimental data.

Upon launching the application, participants provided informed consent online by acknowledging statements regarding voluntary participation, anonymity, and the use of data for scientific purposes. Participants then completed a short demographic form including age and sex. The application sequentially presented reference images of the three target buildings. Each image, captured from the observation point, displayed the building with a colored circular indicator marking the target. Participants were instructed on the identity and position of each building prior to testing to ensure accurate recognition. For each target, participants entered their estimated distance in meters. After submission, the system provided immediate corrective feedback (too short/too long), guiding perceptual recalibration. Participants repeated estimations iteratively until the correct value was reached, enabling the analysis of adaptive changes in perception across successive trials.

All individual estimates were recorded for each trial, allowing detailed tracking of participants’ performance across iterations. For each target building, the system automatically computed summary metrics for each object, including the first estimate (FE), the mean of all estimates (ME), the total number of attempts (NA), and the duration of the trial (TD). This approach enabled a fine-grained analysis of adaptive perceptual recalibration while preserving the complete trial-by-trial dataset for subsequent statistical analysis.

### 2.6. Data Analysis

All variables were first examined for normality using the Shapiro–Wilk test, which indicated that none of the distance estimation measures across targets and trials followed a normal distribution. Consequently, non-parametric statistical methods were employed. Descriptive statistics included means, standard deviations, medians, and interquartile ranges (25th percentile, Q1, and 75th percentile, Q3), providing a comprehensive overview of participants’ performance and trial-by-trial variability.

Inferential analyses focused on evaluating feedback-driven changes in estimation accuracy across the three target buildings. Friedman tests were applied to compare repeated measurements, and when significant differences were detected, post hoc pairwise comparisons were conducted using the Wilcoxon signed-rank test with Bonferroni correction to control for multiple comparisons. Statistical significance was set at α = 0.05, and all analyses were conducted using IBM SPSS 26.0 software (IBM Corp., Armonk, NY, USA) [23].

## 3. Results

Section 3 presents the quantitative outcomes of the egocentric distance estimation task conducted under static vista-space conditions. Data are organized according to target distance and iteration sequence, emphasizing the effects of feedback-driven recalibration on perceptual accuracy and task efficiency.

### 3.1. Descriptive Analysis

The final sample analyzed consisted of 133 participants, following the exclusion of 15 respondents due to incomplete task completion or non-reliable performance data. Exclusion criteria included missing responses, internally inconsistent estimates, and extreme initial distance values that were clearly outside the task-relevant spatial scale. Participants had a mean age of 26.3 ± 7.44 years (range: 18–49 years), and the sample included 76% female and 24% male individuals. Descriptive analyses (Table 1) provide a comprehensive overview of participants’ performance across the three target distances. In Table 1, the error of the first estimate (EFE) and the error of the mean estimate (EME) were calculated as the signed differences between the FE and, respectively, the ME and the D, indicating both the magnitude and direction of estimation bias.

### 3.2. Inferential Analysis

Given that all measured variables violated the assumption of normality (Shapiro–Wilk test, *p* < 0.05), nonparametric repeated-measures analyses were applied. Inferential analyses focused on the core performance measures across trials (EFE, EME, NA, and TD). For each variable, a Friedman test was performed to evaluate within-subject differences across the three target distances (134 m, 575 m, and 1463 m), allowing identification of systematic, scale-dependent variations in perceptual–cognitive calibration. Omnibus Friedman test statistics for all dependent variables are summarized in Table 2.

All analyzed variables (EFE, EME, NA, and TD) showed statistically significant differences in the Friedman test. In terms of effect size, Kendall’s W indicated small effects for all variables, suggesting modest improvements in perceptual accuracy, estimation efficiency, and task duration. These interpretations follow the conventional thresholds for Kendall’s W (0.1 = small, 0.3 = moderate, 0.5 = large) [24]. Pairwise post hoc comparisons were subsequently conducted using Wilcoxon signed-rank tests with Bonferroni-adjusted significance thresholds (Table 3), providing a detailed view of how iterative feedback influenced distance estimation across target distances.

Post hoc comparisons of distance estimation performance, combined with directional patterns observed in the data, revealed systematic effects of target distance on perceptual accuracy and task dynamics.

**Figure 1:**
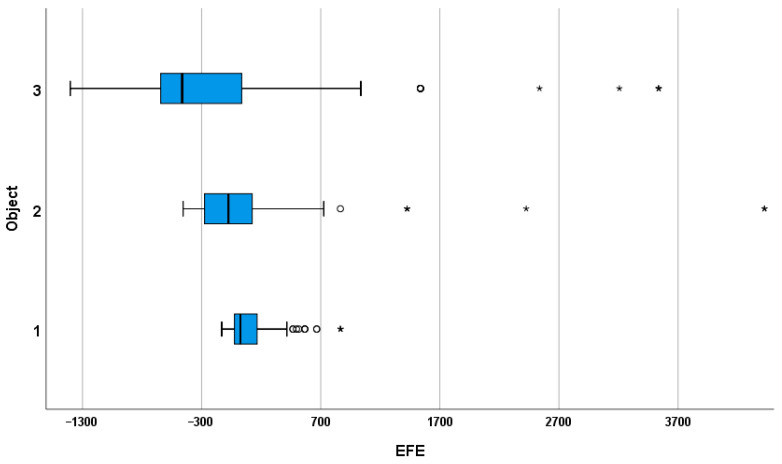
Distribution of error of first estimate (EFE) by object category. Boxplots show median and interquartile range (IQR); whiskers indicate values within 1.5 × IQR; circles and asterisks mark mild and extreme outliers.

Median EFE values showed a progressive shift with increasing target distance (Object 1 = 26 m; Object 2 = −75 m; Object 3 = −463 m). This trend indicates that participants slightly overestimated near targets but increasingly underestimated distances as targets became farther. All pairwise Wilcoxon comparisons were significant (*p* < 0.0167), confirming a systematic growth in perceptual bias with distance. A detailed visualization of EFE distributions across the three target distances is presented in Figure 1.

**Figure 2:**
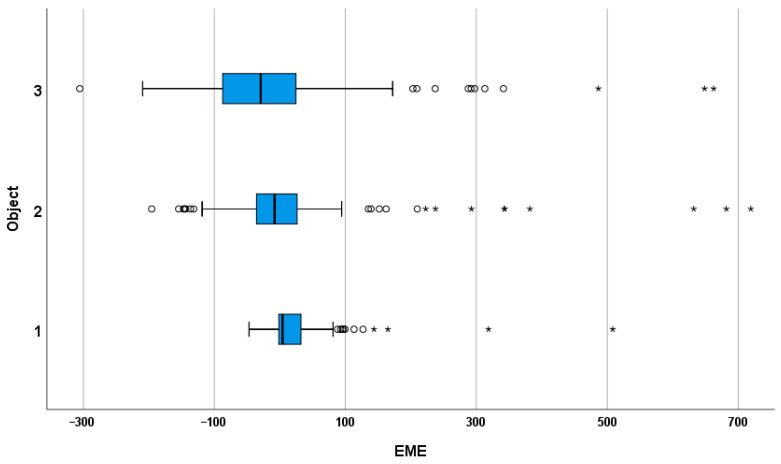
Distribution of error of mean estimate (EME) by object category. Boxplots show median and interquartile range (IQR); whiskers indicate values within 1.5 × IQR; circles and asterisks mark mild and extreme outliers.

Median EME values followed a distance-dependent pattern, with near targets slightly overestimated (Object 1 = 4.11 m) and more distant targets increasingly underestimated (Object 2 = −8 m; Object 3 = −29.27 m). The differences across all target distances were statistically significant (*p* < 0.0167), reflecting the persistence of a compressed distance perception for farther objects despite iterative feedback and recalibration. Figure 2 illustrates the distribution of EME values across object conditions.

**Figure 3:**
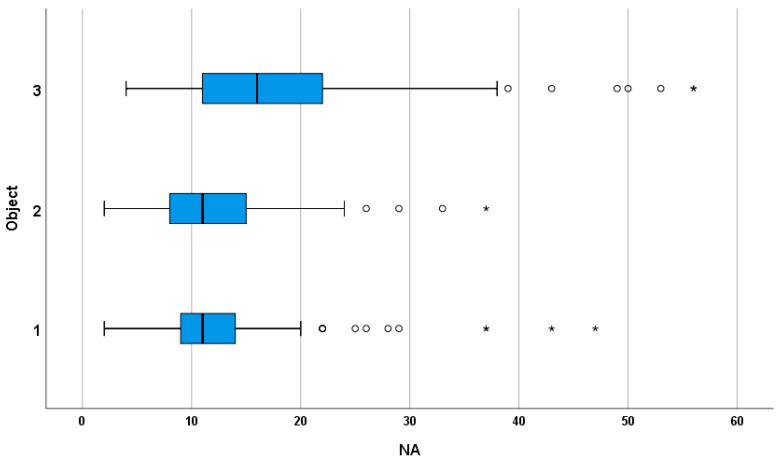
Distribution of number of attempts (NA) by object category. Boxplots show median and interquartile range (IQR); whiskers indicate values within 1.5 × IQR; circles and asterisks mark mild and extreme outliers.

Median NA values were stable for Object 1 and Object 2 (11) but increased for Object 3 (16), indicating that more iterations were needed for the most distant target. Only comparisons involving Object 3 were statistically significant (*p* < 0.0167). The corresponding distribution of NA values across target distances is shown in Figure 3.

**Figure 4:**
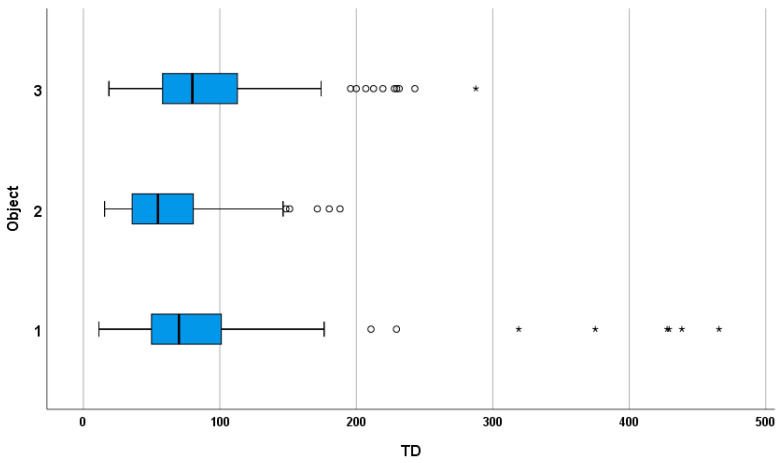
Distribution of trial duration (TD) by object category. Boxplots show median and interquartile range (IQR); whiskers indicate values within 1.5 × IQR; circles and asterisks mark mild and extreme outliers.

Median TD were 70.02 s (Object 1), 54.63 s (Object 2), and 79.91 s (Object 3). Post hoc comparisons indicated significant changes from Object 1 to 2 and from Object 2 to 3, but not from Object 1 to 3, suggesting a non-monotonic pattern in temporal effort across targets. Figure 4 depicts the distribution of trial duration (TD) values for the three object categories.

Overall, these analyses reveal systematic, distance-dependent effects. Estimation errors (EFE, EME) increased with target distance, indicating stronger underestimation at farther scales. Iterative feedback-maintained estimation stability but did not counteract the distance-related bias. Task-related measures (NA, TD) also rose with distance, reflecting greater cognitive and temporal demands.

## 4. Discussion

The aim of the present study was to investigate how iterative, feedback-driven recalibration influences static egocentric distance estimation in vista-space conditions. In line with this objective, the data reveals a systematic underestimation of more distant objects, despite iterative feedback, indicating that perceptual–cognitive mechanisms in visual distance perception are strongly influenced by spatial scale and the inherent limitations of mental representations. At the same time, the feedback-driven recalibration highlights perceptual plasticity and the cognitive system’s ability to adjust estimates based on corrective information.

The following discussion is organized around the main performance variables analyzed in this study (EFE, EME, NA, and TD). For each variable, we focus on the observed patterns across target distances, interpret the findings in relation to existing literature, and highlight their implications for the perceptual–cognitive mechanisms underlying static egocentric distance estimation.

Analysis of the EFE revealed a systematic distance-dependent pattern. Participants slightly overestimated the near target (Object 1, median EFE = 26 m), underestimated the intermediate target (Object 2, median EFE = −75 m), and strongly underestimated the most distant target (Object 3, median EFE = −463 m). Inferential analysis confirmed significant differences across distances (Friedman test, *p* < 0.001), and post hoc pairwise comparisons indicated significant differences between all target pairs (*p* < 0.017).

These results indicate that egocentric distance perception in vista space is not uniform and reflects two partially dissociable processing stages. At an early perceptual level, static egocentric distance judgments are constrained by scale-dependent encoding limitations, such as distance compression for far targets, particularly under conditions of limited depth cues [5,9]. At the same time, other studies have reported overestimation of distant targets as a function of observer position, target size, and environmental context [4], underscoring the variability of early perceptual encoding depending on situational factors. The pronounced underestimation observed for the farthest object, therefore, likely originates from initial perceptual encoding biases related to spatial scale and visual information availability. Importantly, these early perceptual limitations provide the baseline upon which later cognitive processes operate. Subsequent adjustments observed across iterative trials reflect higher-order cognitive correction mechanisms, through which participants use feedback to monitor error, refine hypotheses, and progressively recalibrate their distance estimates without fully eliminating the underlying perceptual bias.

In our experimental paradigm, participants sequentially estimated objects arranged by increasing distance, initially overestimating the nearest target and subsequently underestimating the more distant ones. Given that the participant sample predominantly consisted of young adults, their perceptual responses align with prior findings showing a systematic underestimation of egocentric distance in younger observers [25]. This pattern highlights the interplay between sensory encoding and higher-order cognitive processes [26], particularly under iterative feedback conditions, where top-down mechanisms such as hypothesis testing and internal spatial adjustment can modulate perceptual estimates, occasionally producing overcorrection or cognitive overshoot effects.

The shift from abstract expectations to increasingly precise perceptual judgments mirrors similar dynamics in attention allocation, where conceptual guidance precedes reliance on perceptual information as experience accumulates [27]. The iterative recalibration thus reflects a dynamic integration of perceptual input and cognitive monitoring in the spatial placement of objects, consistent with evidence that individuals’ spatial cognition is shaped by heterogeneous perceptual and cognitive processing [28,29].

In a similar manner to the EFE results, the analysis of EME revealed a comparable distance-dependent pattern. Participants slightly overestimated the closest target and progressively underestimated the more distant ones. Median EME values were 4.11 m for the closest, −8 m for the intermediate, and −29.27 m for the far target. All differences reached statistical significance (Friedman test *p* < 0.001, Wilcoxon post hoc *p* < 0.017). This parallelism between EFE and EME patterns indicates that iterative feedback partially mitigates perceptual bias but does not fully correct the underlying tendency for distance compression in vista space. These findings are consistent with prior evidence showing that egocentric distance judgments are systematically biased under both full-cue and reduced-cue conditions, with near distances often overestimated and far distances underestimated [30,31]. The persistence of this bias despite feedback suggests that stable perceptual priors and early sensory encoding continue to influence egocentric scaling, while higher-order cognitive monitoring may engage in partial error correction.

Furthermore, these findings align with evidence that cognitive factors, including response calibration and task instructions, can modulate verbal distance reports without altering the underlying perceptual representation [32]. Together, these results suggest that EME reflects the dynamic interplay of sensory encoding and cognitive monitoring, with perceptual priors interacting with top-down recalibration during iterative estimation.

The pattern of EME observed in our study can be interpreted through the lens of iterative feedback as a cognitive search process within bounded intervals. Once participants established an initial under- and overestimation range for a target, subsequent attempts involved refining the estimate, effectively narrowing the interval between known lower and upper bounds. This behavior shows a functional resemblance to a binary search algorithm, in the sense that each iterative adjustment tends to reduce the remaining uncertainty according to the scale of the interval [33,34], although this comparison is intended as a heuristic analogy rather than as evidence of a formally implemented cognitive algorithm.

In this framework, EME reflects not only perceptual encoding but also patterns of adjustment that are consistent with feedback-guided, higher-order cognitive processing, without implying an ideal observer or formally optimal performance. The iterative recalibration remains consistent with principles of Bayesian updating and progressive uncertainty reduction, whereby each attempt incorporates prior information and corrective feedback to improve accuracy [35,36]. Consequently, the number of attempts and the direction of corrections observed in our data can be understood as emergent properties of adaptive perceptual–cognitive adjustment rather than of an explicit optimal search mechanism.

Analogous to the EME analysis, the distribution of NA reflects the interaction between initial sensory–perceptual evaluation and the cognitive process of iterative recalibration. Once participants established an initial over- and underestimation range for each target object, each successive attempt involved adjusting the estimate, progressively reducing the remaining uncertainty. This strategy resembles a binary search algorithm, with each iteration decreasing uncertainty according to a logarithmic model of the interval [33,34,35].

Considering the scale and magnitude of the targets, a normative reference can be derived from the minimal number of iterations expected under an idealized binary search framework. For the nearest object (D = 134 m), such a reference corresponds to approximately [log_2_(134)] ≈ 8 iterations, for the intermediate object (D = 575 m), [log_2_(575)] ≈ 10 iterations, and for the farthest object (D = 1463 m), [log_2_(1463)] ≈ 11 iterations. Importantly, these values are not interpreted as predictions of actual cognitive processing, but as theoretical benchmarks for efficient interval reduction. In comparison, the observed median numbers of attempts (Object 1 = 11, Object 2 = 11, Object 3 = 16) suggest convergence behavior that is broadly compatible with progressive uncertainty reduction, while also reflecting additional variability introduced by perceptual noise, sensory imprecision, and non-optimal iterative adjustments. Calculating a simple efficiency index as the ratio of ideal to observed iterations yields 73% for Object 1, 91% for Object 2, and 69% for Object 3. These calculations provide preliminary computational validation, with the idealized binary search serving as a simple normative model and suggest that future studies could extend this approach using formal Bayesian or probabilistic models to further quantify iterative feedback and uncertainty reduction.

Inferential analysis confirmed a significant effect of target distance on NA (Friedman test, *p* < 0.001). Post hoc Wilcoxon comparisons revealed that the farthest object required significantly more attempts than both the nearest and intermediate objects (*p* < 0.017), while no significant difference was observed between the nearest and intermediate targets.

Thus, NA reflects both the perceptual difficulty of distance estimation and the implementation of an efficient cognitive strategy, combining sensory encoding with iterative feedback to refine estimates. The higher number of attempts for the farthest object highlights both the increased initial uncertainty and the greater complexity of adjustments required to converge on the correct value. This pattern suggests that the cognitive system operates as an approximate ideal observer, exploiting feedback to optimize successive estimates within bounded intervals, consistent with multidimensional binary search principles [37].

In parallel with the analysis of NA, TD provides complementary insight into the cognitive demands of iterative distance estimation. The observed trial duration (TD) may reflect the cognitive demands associated with iterative distance estimation. This is similar to findings in temporal discrimination tasks where task difficulty and attentional capacities influence response times [38,39,40]. More generally, TD aligns with literature showing that response times can serve as indicators of cognitive effort and task complexity [41,42]. While a higher number of attempts naturally increases the total trial time, TD also reflects the time participants spend evaluating, planning, and executing each adjustment. In our data, TD showed a non-monotonic pattern across targets, with median values of 70.02 s, 54.63 s, and 79.91 s for the near, intermediate, and far targets, respectively, and post hoc comparisons indicated significant differences between Object 1 and 2, and between Object 2 and 3 (Friedman test, *p* < 0.001; Wilcoxon post hoc, *p* < 0.017).

Therefore, we conclude that the farthest target required both more iterations and longer deliberation per attempt. This pattern suggests that TD captures both interval refinement effects similar to a binary search and the inter-individual differences in cognitive processing speed, decision-making strategies, and allocation of attentional resources during each iterative adjustment. Such patterns of prolonged deliberation are consistent with prior evidence linking response times to cognitive effort and decision complexity [43,44]. Hence, TD serves as an index of the temporal cost of iterative recalibration, complementing NA by reflecting the number of adjustments and the depth of cognitive engagement per iteration. The observed non-monotonic modulation across targets further highlights that TD emerges from a combination of perceptual uncertainty, task complexity, and individual cognitive strategies, consistent with a model of feedback-driven optimization in egocentric distance estimation.

Beyond these variable-specific interpretations, it is also important to consider the consistency of the observed effects across participants. Although Kendall’s W values indicate small effect sizes, their consistency across participants and conditions is theoretically meaningful. In the context of iterative recalibration, such small but reliable effects reflect stable convergence patterns rather than large inter-individual differences, a pattern that is expected in large samples and informative for modeling gradual perceptual–cognitive adjustment. These stable group-level patterns can coexist with meaningful individual differences in calibration strategies, highlighting the nuanced interplay between shared and idiosyncratic aspects of perceptual–cognitive recalibration.

These findings can be situated within broader theoretical accounts of egocentric distance perception. Reviews of egocentric distance estimation in virtual environments indicate systematic underestimation and emphasize that perceived distance is shaped by multiple interacting perceptual, task-related, and observer-dependent factors, with feedback primarily supporting recalibration rather than eliminating early perceptual biases [45]. In parallel, ongoing debates on action-specific influences underscore the importance of distinguishing changes in perceptual encoding from post-perceptual decision and response processes [15]. Within this framework, the present results are more consistent with feedback-driven cognitive correction and response recalibration than with direct modulation of early visual perception. Importantly, these broader implications for cognitive bias and information integration likely reflect distributed interactions across perceptual, decision-related, and higher-order control processes, rather than a single unified mechanism.

Several limitations of this study should be acknowledged. The static vista-space paradigm may not fully capture dynamic cues present in real-world distance perception. In addition, the relatively young and predominantly female (76%), university-based sample tested under specific outdoor conditions may limit the generalizability of the findings. Previous research has shown that spatial perception and distance-related judgments are modulated by individual factors such as age and sex, with systematic differences observed across demographic groups [46]. In the specific context of egocentric distance perception, differences in accuracy and precision have been reported between age and sex groups, as well as in their sensitivity to environmental context [47]. Moreover, environmental conditions and available visual cues have been shown to influence perceived distance, affecting both accuracy and precision across different settings [48]. Future studies should therefore include more age- and sex-diverse samples and systematically examine environmental variability or long-term familiarity with large-scale spaces to better characterize how these factors interact with perceptual–cognitive recalibration processes.

From a design perspective, the absence of a no-feedback control condition and the fixed ordering of target distances also represent methodological constraints. Although this structure was chosen to preserve ecological coherence and isolate feedback-driven recalibration across increasing spatial scales, future work should incorporate randomized target sequences and control conditions to further disentangle order effects from feedback-specific mechanisms.

Finally, the use of a web-based digital application may introduce cognitive or motor-related biases affecting iterative recalibration [49]. While the binary-search framework provides a useful conceptual reference, actual estimation strategies likely reflect the combined influence of perceptual noise, attentional variability, and individual differences, and were not intended to model fully controlled environmental conditions.

In sum, the present findings highlight the persistence of scale-dependent biases in egocentric distance estimation despite iterative feedback, framing recalibration as a cognitive search process akin to binary search. This approach clarifies the interplay between perceptual encoding and higher-order cognitive strategies and provides a novel, quantitative framework for modeling human estimation in constrained environments. Future research could extend these methods to dynamic settings, multi-sensory integration, and applied contexts such as navigation, virtual reality, or robotic–human interfaces, offering practical avenues to enhance spatial cognition, training, and adaptive feedback systems.

## 5. Conclusions

The present study identifies systematic, scale-dependent patterns in egocentric distance estimation, showing that iterative feedback can partially attenuate, but not eliminate, perceptual biases associated with distance compression. Inferential analyses confirmed robust effects across all core performance variables (EFE, EME, NA, and TD), supporting the view that recalibration emerges from the interaction between sensory encoding constraints and higher-order cognitive monitoring, instead of forming a fully optimal correction.

The observed adjustment dynamics are consistent with heuristic models of feedback-guided estimation and progressive uncertainty reduction, rather than with a fully optimal or mechanistically specified ideal observer strategy. We note that the binary search analogy is intended as a conceptual heuristic for understanding these dynamics and does not imply a mechanistic or algorithmic implementation. Within this framework, the mobile digital paradigm employed here serves as a flexible experimental platform for examining perceptual–cognitive calibration under controlled conditions. While the present findings are constrained to the experimental context, they contribute to broader theoretical perspectives on cognitive bias, perceptual calibration, and system-level information integration. These insights provide a conceptual foundation for future work in virtual environments, spatial training, and human–machine interaction, without implying direct clinical or translational generalization.

## Figures and Tables

**Table 1:** Descriptive summary of participant performance variables across the three target distances (*n* = 133).

	Variable	FE (m)	EFE (m)	ME (m)	EME (m)	NA	TD (s)
Object 1 (D = 134 m)	Mean ± SD	247.39 ± 211.87	113.39 ± 211.87	157.81 ± 60.89	23.81 ± 60.89	12.58 ± 6.95	92.03 ± 79.99
	Median	160	26	138.11	4.11	11	70.02
	95% CI	150–200	16–66	136.05–141.33	2.05–7.33	11–11	63.69–76.45
	Q1–Q3	110–300	−24–166	132.33–167.39	−1.68–33.39	9–14.5	49.95–101.45
Object 2 (D = 575 m)	Mean ± SD	636.81 ± 688.52	61.81 ± 688.52	593.38 ± 136.20	18.38 ± 136.20	12.34 ± 6.08	62.46 ± 34.65
	Median	500	−75	567	−8	11	54.63
	95% CI	400–512.68	−175–−62.32	559.8–575.85	−15.2–0.85	11–11	48.23–60.98
	Q1–Q3	300–710	−275–135	538.58–601.44	−36.43–26.44	8–15	35.54–80.91
Object 3 (D = 1463 m)	Mean ± SD	1333.23 ± 890.66	−129.77 ± 890.66	1469.90 ± 232.71	6.90 ± 232.71	18.49 ± 10.30	93.60 ± 52.66
	Median	1000	−463	1433.73	−29.27	16	79.91
	95% CI	1000–1000	−463–−463	1420.9–1450.3	−42.1–−12.7	16–16	72.05–87.23
	Q1–Q3	810–1500	−653–37	1375.40–1488.10	−87.60–25.10	11–22.5	57.77–115.34

Note—FE: First estimate; EFE: error of first estimate; ME: mean estimate; EME: error of mean estimate; NA: number of attempts; TD: trial duration; D: true distance; SD: standard deviation; Q1: first quartile (25th percentile); Q3: third quartile (75th percentile); 95% CI: 95% bootstrap confidence interval for the median; *n*: number of participants. All values represent participant-level aggregated data per target.

**Table 2:** Summary of Friedman test statistics for distance estimation variables across the three target distances.

Variable	χ^2^ (df = 2)	Asymp. Sig.	Kendall’s W	Interpretation
EFE	59.73	<0.001	0.225	Significant
EME	32.66	<0.001	0.123	Significant
NA	51.36	<0.001	0.193	Significant
TD	41.07	<0.001	0.154	Significant

Note—χ^2^: Friedman chi-square test statistic; df: degrees of freedom; EFE: error of first estimate; EME: error of mean estimate; NA: number of attempts; TD: trial duration; Asymp. Sig.: two-tailed *p*-value; W: Kendall’s coefficient of concordance, representing effect size (small = 0.1, moderate = 0.3, large = 0.5).

**Table 3:** Post hoc pairwise comparisons of distance estimation variables using Wilcoxon signed-rank tests with Bonferroni correction.

Variable	Pairwise Comparison	Z (Wilcoxon)	Asymp. Sig.	*p*-Value Bonferroni	Interpretation
EFE	Object 1 vs. Object 2	−4.425	0.001	0.0167	Significant
	Object 1 vs. Object 3	−5.112	0.001	0.0167	Significant
	Object 2 vs. Object 3	−4.619	0.001	0.0167	Significant
EME	Object 1 vs. Object 2	−3.134	0.002	0.0167	Significant
	Object 1 vs. Object 3	−4.110	0.001	0.0167	Significant
	Object 2 vs. Object 3	−2.377	0.016	0.0167	Significant
NA	Object 1 vs. Object 2	−0.167	0.868	0.0167	n.s.
	Object 1 vs. Object 3	−6.367	0.001	0.0167	Significant
	Object 2 vs. Object 3	−6.127	0.001	0.0167	Significant
TD	Object 1 vs. Object 2	−4.389	0.001	0.0167	Significant
	Object 1 vs. Object 3	−2.054	0.04	0.0167	n.s.
	Object 2 vs. Object 3	−6.139	0.001	0.0167	Significant

Note—EFE: error of first estimate; EME: error of mean estimate; NA: number of attempts; TD: trial duration; Z: Wilcoxon test statistic; Asymp. Sig.: two-tailed *p*-value; *p*-value Bonferroni: significance threshold adjusted for 3 pairwise comparisons (α = 0.05/3 = 0.0167); n.s.: nonsignificant.

## Data Availability

The data are available on request from the corresponding author. All data relevant to the study are included in the article.

## Abbreviations

The following abbreviations are used in this manuscript:

FE	First Estimate
EFE	Error of First Estimate
ME	Mean Estimate
EME	Error of Mean Estimate
NA	Number of Attempts
TD	Trial Duration
D	True Distance
IQR	Interquartile Range
